# Literary Notes

**Published:** 1907-08

**Authors:** 


### LITERARY NOTES.

OUR OWN FOLKS is the title of a four page quarto leaflet published by D. Appleton and Company, New York, in the interests of the people of the house of Appleton. The issue for July is the seventh of the series and contains portraits of Messrs. Randolph, Davis, Bryant and Pridgen, managers of agencies respectively in the cities of New York, San Francisco, Pittsburg, and Atlanta. It contains much interesting material relating to the medical book trade and should be of value in promoting the sale of the publications of the celebrated house from which it issues.

THE SAINT LOUIS MEDICAL REVIEW has ceased publication as a weekly medical journal, but will be continued in a new series as a monthly magazine. It is understood that the accomplished editor, Dr. Kenneth W. Millican, has joined the editorial staff of the Journal of the American Medical Association.

THE INTERSTATE MEDICAL JOURNAL (Saint Louis) announces the purchase of the *Saint Louis Courier of Medicine,* one of the oldest medical journals in the West, and its consolidation with the *Interstate* on July 1. This merger removes from the field an old and highly esteemed contemporary, and its consolidation with the *Interstate* adds strength and prestige to that periodical. This is the fourth medical journal that has been purchased and absorbed by the *Interstate* during the past few years.

